# Gastrointestinal challenges in nephropathic cystinosis: clinical perspectives

**DOI:** 10.1007/s00467-023-06211-6

**Published:** 2024-02-23

**Authors:** Mark W. Joseph, Deborah R. Stein, Adam C. Stein

**Affiliations:** 1 Pediatric Nephrology, Oregon Health & Science University and OHSU Doernbecher Children’s Hospital, Portland, OR USA; 2 Pediatric Nephrology, Harvard Medical School and Boston Children’s Hospital, Boston, MA USA; 3 Gastroenterology, Northwestern University and Northwestern Medicine, Chicago, IL USA

**Keywords:** Nephropathic cystinosis, Gastrointestinal side effects, Cysteamine, Cystine-depleting therapy, Reflux, Dysphagia

## Abstract

Gastrointestinal (GI) sequelae, such as vomiting, hyperacidity, dysphagia, dysmotility, and diarrhea, are nearly universal among patients with nephropathic cystinosis. These complications result from disease processes (e.g., kidney disease, cystine crystal accumulation in the GI tract) and side effects of treatments (e.g., cysteamine, immunosuppressive therapy). GI involvement can negatively impact patient well-being and jeopardize disease outcomes by compromising drug absorption and patient adherence to the strict treatment regimen required to manage cystinosis. Given improved life expectancy due to advances in kidney transplantation and the transformative impact of cystine-depleting therapy, nephrologists are increasingly focused on addressing extra-renal complications and quality of life in patients with cystinosis. However, there is a lack of clinical data and guidance to inform GI-related monitoring, interventions, and referrals by nephrologists. Various publications have examined the prevalence and pathophysiology of selected GI complications in cystinosis, but none have summarized the full picture or provided guidance based on the literature and expert experience. We aim to comprehensively review GI sequelae associated with cystinosis and its treatments and to discuss approaches for monitoring and managing these complications, including the involvement of gastroenterology and other disciplines.

## Introduction

Nephropathic cystinosis is a rare autosomal recessive lysosomal storage disorder caused by pathogenic variants in the *CTNS* gene on chromosome 17. Its estimated incidence is 1 in 100,000 to 200,000 live births [1]. Impaired transport of cystine out of lysosomes results in cystine accumulation throughout the body, reaching toxic levels and precipitating as intralysosomal crystals. Patients are typically diagnosed in the first year of life, presenting most commonly with Fanconi syndrome, failure to thrive, and rickets [1, 2]. The kidneys are the first and most severely impacted organ [1, 3]. Without intervention, patients typically progress to kidney failure, requiring dialysis or kidney transplant by 10 years of age [4]. After kidney transplant, cystine continues to accumulate in other tissues [1, 3]. Cystine-depleting therapy (CDT) reduces intralysosomal cystine and is currently the only available disease-modifying treatment [5]. Cysteamine therapy and kidney transplantation have prolonged survival and changed the disease trajectory from a fatal kidney disease to a chronic multisystemic condition [3, 6]. Accordingly, it has become essential to understand and manage extra-renal complications, including the heavy burden of gastrointestinal (GI) complications, for improved patient care and outcomes [7–9].

### Overview of GI concerns in cystinosis

GI complications are universal among patients with cystinosis, occurring at virtually every disease stage [7, 9, 10]. Oral cysteamine therapy is ulcerogenic and known to cause GI symptoms via gastrin release and gastric acid hypersecretion [11–13]. Additionally, cystine accumulation and crystal deposition cause tissue inflammation and damage, resulting in a variety of further GI consequences [12]. Current understanding of GI manifestations has been informed by survey data and patient experiences. Common GI complications across survey and clinical studies include vomiting, nausea, poor appetite, dysphagia, diarrhea, hepatomegaly, abdominal pain, reflux, and dysmotility (Table 1) [6, 8–12, 14–20]. Given the rarity of cystinosis, studies cited throughout this review are mostly small and almost exclusively observational, with great heterogeneity in era and patient demographics/characteristics, making it difficult to interpret differences based on factors such as patient age and availability and adequacy of CDT. In a 1998 North American patient registry study, Elenberg et al. first established that GI symptoms are common and occur earlier than previously recognized. According to responses from 70 patients (aged 1–37 years) and caregivers, 93% of patients had GI symptoms at initial presentation, and lifetime prevalence of GI complications was 100% [9]. In a 2011 Cystinosis Research Network (CRN) survey of 72 caregivers, 40% reported that GI complications impact their children’s health, second only to kidney complications [21]. CRN’s subsequent 2016 survey of 52 adults found that a high percentage experienced GI symptoms weekly: 54% reported swallowing difficulties, 38% nausea, 21% loss of appetite, 19% diarrhea, and 15% vomiting [14]. In another CRN survey, < 20% of adults reported seeing a gastroenterologist (*n* = 74) [14].

**Table 1:** Prevalence of common GI complaints in cystinosis clinical and survey studies by patient age and study year^a^

Study year, type, and population	Swallowing dysfunction	Vomiting	Nausea	Acid reflux/esophagitis	Ulcer	Poor appetite	Abdominal pain	Dysmotility/ pseudo-obstruction	Diarrhea	Constipation	Hepatomegaly	Splenomegaly	Increased portal vein velocity
All ages													
1998 survey study [9] (*N* = 70; aged 1–37 years)^b^	13^c^–41%^d^	83%^e^		6–21%	3%	71%	50%	6–10%	57%^e^		-	-	-
2005 clinical and survey study [18] (*N* = 101; aged 6–45 years)^f^	24^c^–74%^d,g^	-	-	-	-	-	-	-	-	-	-	-	-
2022 clinical study [20] (*N* = 55; aged 3–41 years)^h^	45%^c^	-	-	-	-	-	-	-	-	-	-	-	-
Children													
2001 clinical and survey study [19] (*N* = 22; aged 2–16 years)^i^	33^c^^,j^–65%^d,k^	55–65%^d,k^	-	-	-	-	-	-	-	-	-	-	-
2003 clinical study [12] (*N* = 11; aged 2–12 years)^l^	-	-	-	18%	-	-	-	-	-	-	-	-	-
2005 clinical study [11] (*N* = 12; aged 2–10 years)^m^	-	-	-	17%	8%	-	-	-	-	-	-	-	-
2009 clinical study [10] (*N* = 23; aged 1–13 years)^n^	4%^d^	70%	9%	-	-	17%	13%	-	26%	17%	35%	22%	-
2020 clinical study [8] (*N* = 21; aged 1–29 years)^o^	30%^c,p^	-	-	32%^q^	-	-	-	-	-	-	74%^q^	53%^q^	58%^q^
Adults													
2007 clinical study [15] (*N* = 100; aged 18–45 years)^r^	60%^c,s^	-	-	-	-	-	-	-	-	-	-	-	-
2012 clinical study [6] (*N* = 86; aged 15–50 years)^t^	20%^c^	-	-	-	-	-	-	-	-	-	-	-	-
2016 survey study [14] (*N* = 52; aged 18– > 56 years)^u^	54%^d^	15%^v^	38%^v^	-	-	21%^v^	-	-	19%^v^	-	-	-	-
2020 clinical and survey study [17] (*N* = 20; aged 20–64 years)^w^	30^c^–60%^d^	-	-	-	-	-	-	-	-	-	-	-	-
2022 clinical study [16] (*N* = 19; aged 18–34 years)^x^	11%^c^	-	42%	16%	-	-	42%	-	11%	-	21%^e^		6%^j^

- GI complication not examined, *GI* gastrointestinal

^a^Study era, patient demographics and characteristics (including availability and adequacy of oral cysteamine therapy), and outcome measures were highly variable

^b^Twenty percent of respondents had undergone kidney transplant, 3% were on dialysis, and 7% reported renal insufficiency not yet requiring dialysis

^c^Based on clinical assessment

^d^Self-/caregiver report

^e^Combined prevalence

^f^Seventy-one percent of patients received cysteamine therapy (mean duration, 7 years), and 80% had undergone kidney transplant

^g^Based on a subgroup of 78 respondents (aged 9–45 years)

^h^All patients received cysteamine therapy, 36% of patients had undergone kidney transplant, and 7% were on dialysis

^i^Eighty-two percent of patients received cysteamine therapy, and 9% had undergone kidney transplant

^j^Based on assessment of 18 patients

^k^Parent reports for 20 patients

^l^No patients had undergone kidney transplant, and all patients were on cysteamine therapy (mean leukocyte cystine level, 1.2 nmol ½ cystine/mg protein)

^m^No patients had undergone kidney transplant, and all patients were on cysteamine therapy (mean leukocyte cystine level, 0.95 nmol ½ cystine/mg protein)

^n^All patients had renal tubular acidosis, and 74% had chronic kidney failure

^o^Median age at cystinosis diagnosis was 1.3 years (range, 0.4–14 years), and mean follow-up after diagnosis was 9.5 years (range, 0.5–20 years). At the time of study, 41% of patients had kidney failure, of whom 56% received a kidney transplant and 44% were on dialysis. All patients had periods without cysteamine treatment

^p^Based on assessment of 20 patients

^q^Based on assessment of 19 patients

^r^Ninety-two percent of patients had undergone kidney transplant, 61% received cysteamine therapy for < 8 years (mean, 2 years), and 39% received cysteamine therapy for ≥ 8 years (mean, 15 years)

^s^Based on assessment of 97 patients

^t^Seventy-six percent of patients had undergone kidney transplant, and 87% received cysteamine therapy (mean duration, 17 years)

^u^Eighty-nine percent of respondents had undergone kidney transplant, and 90% reported being on cysteamine therapy

^v^Percentage of patients reporting these symptoms at least once per week

^w^Ninety percent of patients had undergone kidney transplant, and 80% received cysteamine treatment

^x^Seventy-four percent of patients had undergone kidney transplant, 16% were on dialysis, and all patients were on cysteamine therapy

Upper GI signs and symptoms are especially concerning due to risks of choking and aspiration [6, 7, 15, 18]. Kasimer and Langman’s 2021 systematic literature review reported 40 to 75% prevalence of dysphagia across studies of patients with cystinosis [7]. Dysphagia, anorexia, nausea, and vomiting can be so severe that feeding tubes are needed to improve symptoms, nutrition, and medication administration, with reported use in 11 to 78% of patients [7, 9, 16, 19].

### Effects of GI complications on therapeutic efficacy and patient outcomes

GI symptoms produce many consequences that further impact patient outcomes and quality of life, including inadequate protein-energy intake, which may contribute to low height and weight parameters commonly seen in children with cystinosis [9]. GI complications can also limit social and physical activities, contribute to school/work absenteeism, and worsen mental health [9, 12, 14]. Even less medically severe GI concerns, such as cysteamine-induced halitosis, impact patient activities and treatment success [14]. GI side effects and disease complications can lead to decreased treatment adherence, which reduces circulating and tissue cysteamine levels, leading to further cystine-induced damage [11, 12, 14]. Adherence to cysteamine dosing recommendations can decline considerably in adolescence and adulthood, with GI side effects often cited as problematic in patient surveys [14, 22].

Early recognition and management of GI symptoms can improve or prevent the development of disabling complications and downstream consequences for well-being and therapeutic success [9]. Given the growing recognition of the prevalence and impact of GI complications in cystinosis and the lack of clinical guidance on this topic, we aim to review the available data and discuss clinical approaches to monitoring and managing GI sequelae in patients with cystinosis.

## Multifactorial origins of GI complications in cystinosis

GI complications in cystinosis originate from tissue damage caused by cystine crystal deposition, consequences of kidney disease and related interventions, and side effects of oral cysteamine and other therapies such as immunosuppression following transplant (Table 2) [7, 12]. Though the spectrum of GI issues associated with cystinosis and CDT can present at any age, those arising from Fanconi syndrome and its treatments tend to be more prevalent in young children, while those stemming from kidney transplant, immunosuppression, and long-term cystine accumulation tend to emerge in adolescence and adulthood [9]. Awareness of these overlapping etiologies can help the clinical team assess and address the diverse GI symptoms in patients with cystinosis [7, 9].

**Table 2:** Key contributors to GI complications in cystinosis

GI complications	Cystinosis	Kidney disease			Polypharmacy		
	Cystine accumulation [8, 12]	Fanconi syndrome [13, 37]	CKD/kidney failure [35, 36, 38]	Dialysis [35, 38, 41]	Oral cysteamine [9, 12, 33, 34, 44]	Immuno-suppressants [42, 43]	Supplements/other [9, 39, 40]^b^
Halitosis					X		
Oromotor/swallowing dysfunction	X						
Nausea/vomiting		X	X	X	X	X	X
Anorexia/poor appetite	X	X	X	X	X	X	
Esophagitis/acid reflux			X	X	X	X	X
Gastritis/gastric ulcer				X	X	X	X
Intestinal ulcer				X	X	X	X
Early satiety	X	X	X	X			
Delayed gastric emptying			X		X	X	
Abdominal pain/distension		X	X	X	X	X	X
Dysmotility^a^	X		X	X			X
Diarrhea					X	X	X
Constipation		X	X	X	X	X	
Intestinal ischemia				X			
Intestinal bleeding/erosion/perforation				X	X	X	X
Hepatomegaly/splenomegaly	X						

*CKD* chronic kidney disease, *GI* gastrointestinal, *NSAID* nonsteroidal anti-inflammatory drug

^a^Including pseudo-obstruction/obstruction

^b^Such as citrate, bicarbonate, phosphate, vitamin D, and NSAIDs

### GI effects of cystine crystal accumulation and resultant inflammation

**Fig. 1:**
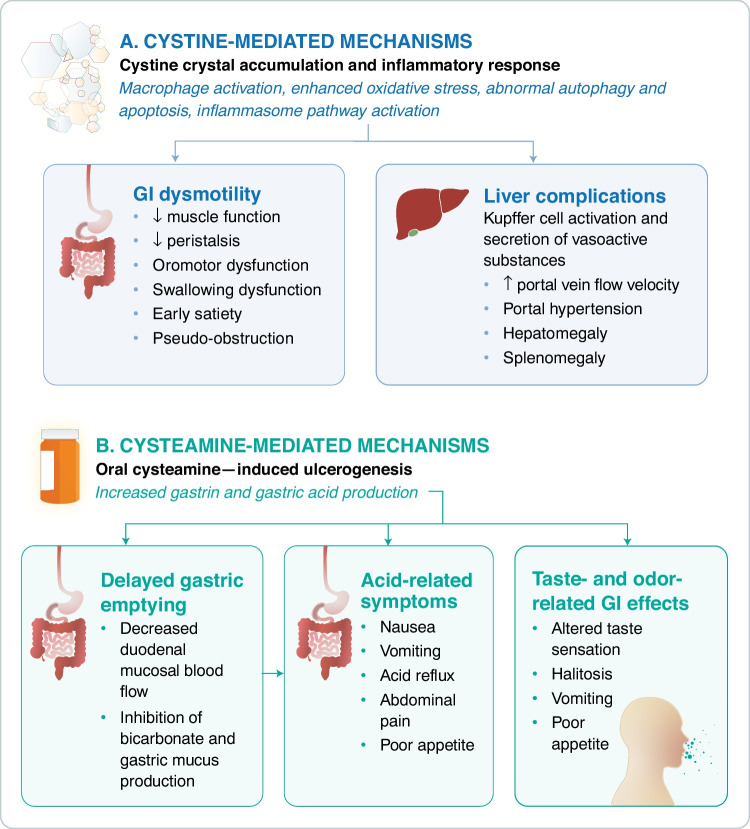
The proposed etiologies of GI complications in cystinosis include a combination of mechanisms related to **A** cystine crystal accumulation and **B** oral cysteamine–induced ulcerogenesis [8, 9, 12, 16, 24, 26–31, 86, 87]. *GI* gastrointestinal

Tissue damage and dysmotility caused by cystine crystal accumulation and associated inflammation are believed to significantly contribute to GI sequelae in cystinosis (Fig. 1) [12]. Dohil et al.’s 2003 and 2005 studies of cysteamine-treated children (*N* = 11 and *N* = 12, respectively; mean age, 6 years) found that > 90% had cystine crystal deposits visible on electron microscopy of upper GI mucosal biopsies, suggesting even long-term CDT with adequate white blood cell (WBC) cystine control does not prevent or deplete all GI tract crystals [11, 12]. The 2005 study found no association between upper GI crystal concentration and age at diagnosis or CDT duration [11]. A later study by Dohil et al. measured crystal load in histiocytes of mucosal biopsies (*N* = 17; mean age, 7 years) and found that longer CDT duration was associated with lower crystal concentrations (*r* = 0.13, *P* = 0.07). After excluding an outlier, mean WBC cystine levels were significantly correlated with crystal concentrations in the stomach and duodenum at all time points (*r* = 0.2, *P* = 0.02). Nonetheless, patients with below-target WBC cystine levels still had detectable crystals [23]. While WBC cystine level is considered the best and most readily available surrogate for tissue cystine accumulation, it represents only short-term treatment adherence and does not reflect variability in tissue-specific rates of cysteamine uptake and cystine depletion [23]. Dohil et al. further established that crystal concentrations vary within the GI tract, with highest to lowest concentrations in biopsies of the rectum/colon (mean, 24), stomach (14), duodenum (10), and esophagus (2) [23]. Researchers have hypothesized that these differences are due to greater cysteamine absorption in the upper GI tract, thus lower mucosal crystal concentrations in those tissues, and/or due to varying rates of cell turnover and protein degradation in different tissues [11, 23].

Beyond the direct harmful effects of cystine crystal deposition, enhanced apoptosis and inflammation have been increasingly implicated as major contributors to disease pathogenesis, including in the GI tract (Fig. 1) [24]. Cystine crystals have been observed inside macrophages of the gastric mucosa and intestinal lamina propria [24, 25]. Moreover, in addition to identifying chronic inactive duodenal inflammation in 33% of cysteamine-treated children, Dohil et al. observed esophageal basal zone hyperplasia and intraepithelial eosinophils in 17 to 18% of these patients [11, 12].

### GI effects of oral cysteamine

Patients with cystinosis are living longer into adulthood due to advances in medical care, including the advent of cysteamine, currently the only available disease-modifying treatment that depletes intralysosomal cystine [3, 5, 6]. Cysteamine is a sulfur-containing compound with a foul odor and taste [7, 9]. It is also ulcerogenic and known to induce GI symptoms via several mechanisms (Fig. 1) [12, 26, 27]. Rat studies demonstrated gastric acid hypersecretion secondary to hypergastrinemia [26, 28, 29] and delayed gastric emptying [30, 31]. Cysteamine-induced gastroparesis may increase gastrin production by prolonging food contact time with gastric mucosa [31]. Within minutes of administration, cysteamine also reduces duodenal mucosal blood flow, hypothesized to alter production of bicarbonate, mucus, and epidermal growth factor from the duodenal submucosa [30]. Rat studies also demonstrated that cysteamine induces duodenal ulcers by increasing intracellular iron uptake in the mucosa and damaging tissue by promoting oxidative stress [32].

While cysteamine dosages used to induce GI ulceration in animal studies are approximately 40 to 60 times greater than those used to treat cystinosis, standard therapeutic dosages in humans have also demonstrated ulcerogenic effects [26, 28, 29]. A 1997 study of four children with cystinosis showed a threefold increase in gastric acid and increased serum gastrin following cysteamine administration [27]. Dohil et al. demonstrated that cysteamine-treated children had fasting baseline hypergastrinemia and high basal gastric acid output, which increased further after cysteamine administration, typically peaking within 60 min and coinciding with symptom onset, even with concomitant acid-suppressing therapy [11, 12].

Product labels for immediate-release (IR) and delayed-release (DR) cysteamine have warnings about GI ulceration and bleeding and recommend monitoring and decreasing dosage for severe symptoms. GI symptoms listed among the most common adverse reactions for these products include vomiting, nausea, anorexia, abdominal pain, gastroenteritis, breath odor, and diarrhea [33, 34].

### GI effects of kidney disease and related interventions

Kidney disease and its treatments also contribute to various GI issues in cystinosis (Table 2). The association between deteriorating kidney function and GI complications is well-established [35]. Several hormones involved in GI motility and regulation of hunger and satiety are significantly elevated in kidney disease, which can result in gastric dysrhythmias and delayed gastric emptying [36]. Feeding problems, dyspepsia, reflux, nausea, vomiting, anorexia, abdominal distension, and constipation are associated with Fanconi syndrome and chronic kidney disease, and therapies for these conditions further compound the risk of GI complications [9, 13, 35–38]. The ulcerogenic potential of indomethacin, sometimes used to improve polyuria and polydipsia, is a concern in cystinosis [9]. Replacement therapies for salts and vitamins, such as citrate, bicarbonate, carnitine, phosphate, calcium, copper, phosphorus, and vitamin D, are associated with GI issues [9, 39, 40], as are dialysis, kidney transplant, and immunosuppressive agents [35, 38, 41–43].

## Key context and strategies for GI complications in cystinosis

Next, we summarize key data and practical strategies based on the literature and clinical experience to inform the assessment and management of GI complications in cystinosis.

### Oral and swallowing complications in cystinosis

#### Halitosis

Oral cysteamine has a foul taste, and a metabolic byproduct dimethyl sulfide (DMS) causes halitosis, which can contribute to altered taste sensation, nausea, decreased appetite, decreased adherence, and psychological distress [9, 22, 44]. Nearly half of surveyed adult patients reported concerns about breath and/or body odor [14]. Because the source of halitosis is exogenous, good oral hygiene does not prevent it [7]. In a small study (*N* = 4), median breath DMS levels were lower after administration of DR versus IR cysteamine, although the difference was not statistically significant (3.5 vs. 7.0 nmol*h/L; *P* = 0.07) [44]. A phase 3b study subset analysis reported that patients on DR cysteamine had a 26% reduction in exhaled DMS versus patients on IR cysteamine (*n* = 20; AUC_0–*t*_, 0.74; 90% CI, 0.52–1.06) [45]. Halitosis may subjectively improve with the use of breath mints, baking soda, chlorophyll, and/or vitamin B2 supplements [2, 46, 47].

#### Swallowing dysfunction

Swallowing-related complications in cystinosis have been attributed to myopathy induced by cystine crystal deposition that causes gradual deterioration of muscle mass and function in the mouth and throat [48]. The frequency and severity of oromotor and swallowing abnormalities generally increase with age [18, 48]; however, problems appear to start early and may not correlate with general disease severity [18, 19].

Like other estimates in cystinosis, the reported prevalence of swallowing dysfunction varies widely (Table 1) [6, 8–10, 14–20]. In 2001, Trauner et al. described a high incidence of feeding and swallowing problems reported by parents of children and adolescents with cystinosis (mean age, 8 years), including gagging (65%), prolonged time to finish meals (55%), choking (45%), and swallowing multiple times for one bite of food (30%). On oromotor examination of 18 patients, all had normal oral phase mechanics, with 83% displaying mild hypotonia [19]. A 2020 study of 21 children in Turkey (mean age, 11 years) found that 20% had esophageal phase swallowing abnormalities, which were more prevalent among children with leukocyte cystine levels ≥ 2 nmol ½ cystine/mg protein versus < 2 nmol ½ cystine/mg protein (57% vs. 14%; *P* = 0.5) [8].

A 2022 German study of 55 children and adults (mean age, 21 years) reported a 45% rate of dysphagia, confirmed via oral contrast swallow and fiberoptic endoscopic evaluation [20]. Sonies et al.’s 2005 study of pediatric and adult patients with cystinosis (mean age, 28 years) evaluated at the National Institutes of Health (NIH) reported swallowing abnormalities in > 50% of patients. In response to a questionnaire (*n* = 78), 26% reported choking, 41% moderate or severe difficulty swallowing solids, and 9% moderate or severe difficulty swallowing liquids. On barium swallow studies (*N* = 101), the oral, pharyngeal, and esophageal swallowing phases were abnormal in 24%, 51%, and 73% of patients, respectively. Of 20 deaths, 9 were due to aspiration or problems related to severe dysphagia [18]. Around the same time, Gahl et al. published a study of 100 consecutive adult patients (mean age, 26 years) examined at the NIH from 1985 to 2006; 60% had documented swallowing abnormalities, and 5 of 33 deaths were attributed to a combination of respiratory and swallowing complications, including aspiration [15]. In both NIH studies, adverse outcomes were associated with shorter cysteamine treatment duration [15, 18]. In a 2020 US study of 20 adults (mean age, 35 years), 60% self-reported dysphagia, confirmed by video fluoroscopy in 30% [17]. Additional analyses of this cohort identified difficulties with tongue control/motion, swallow initiation, laryngeal elevation, and pharyngeal residue in ≥ 50% of patients, including in the absence of advanced myopathy [49].

It is important to mitigate proximal myopathy via cystine control due to the devastating effects of dysphagia on morbidity and mortality in cystinosis and because treatments for dysphagia are very limited (Table 3) [6, 7, 15, 18]. Several studies have linked longer cysteamine treatment to lower rates of myopathy and swallowing dysfunction [6, 15, 18]. In addition to frequent monitoring to ensure good cysteamine adherence and cystine control [6], patients should be surveilled regularly for dysphagia [18]. International consensus-based guidance published in 2022 recommends soliciting patient-reported symptoms (e.g., chewing difficulties, aspiration, dysphagia, excess saliva, weight loss, long mealtimes, respiratory infections) at each visit for adolescents and adults [39].

**Table 3:** Clinical strategies for oromotor and swallowing dysfunction in cystinosis

Monitoring	• At each visit, monitor for dysphagia by patient-reported symptoms (e.g., difficulties with chewing, aspiration, dysphagia, excess saliva, weight loss, long mealtimes, respiratory infections) [39] - Consider use of patient symptom questionnaires^a^ (e.g., Pedi-EAT-10,^b^ EAT-10^c^) [17, 49] • Conduct regular granulocyte cystine level monitoring to ensure good cysteamine adherence and cystine control for mitigation of long-term myopathic processes [13]
Specialist consultation and referral	• Consult with specialists for further evaluations and interventions (e.g., gastroenterologist, neurologist, dietitian, speech therapist, OT, PT, ENT) [39, 50, 51] - Clinical assessments^a^ (e.g., TOMASS-C,^d^ TOMASS,^e^ bedside water swallowing test) [39, 49] - Swallowing studies (e.g., video fluoroscopy, fiberoptic endoscopy evaluation) [39, 47, 49, 51] - Electromyography, if clinically indicated [39, 47, 51] - Pharmacist/polypharmacy consultation [47]
Potential interventions	• Refer to specialists who may consider or implement: - Dietary modifications (e.g., thickening, pureeing foods) [9, 48, 49] - Postural compensations (e.g., food placement at the back of the tongue, chin tuck) [9, 48, 49] - Exercises to reduce risk of aspiration (e.g., laryngeal adduction, voluntary swallowing, muscle activation/strengthening) [9, 48, 49, 52] - Regular physical activity [51] - Tube feeding [7, 9, 13, 51] - Surgical treatment [2, 9]

*EAT-10* Eating Assessment Tool, *ENT* otorhinolaryngologist, *OT* occupational therapist, *Pedi-EAT-10* Pediatric Eating Assessment Tool, *PT* physical therapist, *TOMASS* Test of Masticating and Swallowing Solids, *TOMASS-C* Test of Masticating and Swallowing Solids in Children

^a^No myopathy or dysphagia assessment instruments have been expressly validated in cystinosis. Sadjadi et al. have proposed the feasibility of a cystinosis-specific functional clinical outcome measure to quantify muscle weakness and dysphagia in adults [17]

^b^Ten-item caregiver-administered outcome instrument considered valid and reliable for assessing dysphagia severity and aspiration risk in children with swallowing disorders [53]

^c^Ten-item self-administered outcome instrument considered valid and reliable for assessing dysphagia severity and treatment response in adults with a wide array of swallowing disorders [54]

^d^Validated in children [55]

^e^Validated in adults [55]

Further assessments and interventions for swallowing dysfunction should be considered based on patient-reported concerns. Specialist consultations may include gastroenterologists, neurologists, otorhinolaryngologists, speech pathologists, and physiotherapists [9, 39, 50, 51]. Swallowing tests may be warranted according to clinical criteria [39, 47].

Strategies to reduce aspiration risk include thickening foods to increase sensation, pureeing to facilitate swallowing, placing food closer to the back of the tongue to compensate for tongue weakness, tucking the chin down to open the pharynx, exercises in laryngeal adduction to protect the airway, and voluntary swallowing [9, 48, 49]. Muscle training may help; a study of 20 adults with cystinosis found modest improvements in swallowing measures among patients with more severe dysphagia after 5 weeks of using a handheld device to improve muscle activation for swallowing and protecting/clearing the airway [52]. Tube feeding and/or surgery may be needed in more advanced cases [2, 7, 9, 13].

### Upper GI complications in cystinosis

Various upper GI disorders, caused by cystine accumulation and treatments, are prominent among patients of all ages. Cystinosis cohort and questionnaire studies have reported relatively high rates of vomiting (15–70%), particularly in children; nausea (9–42%); acid reflux and/or esophagitis (6–32%); and peptic ulcer (3–8%) (Table 1) [8–12, 14, 16, 19]. Many of these symptoms are closely linked with poor appetite, delayed gastric emptying, and early satiety, which negatively impact therapeutic success, nutrition, growth, and development [7, 9].

Oral cysteamine can potently increase gastric acid production, and cystine accumulation is hypothesized to delay gastric emptying via crystal-induced tissue damage [12]. Delayed gastric emptying increases contact time between food and gastric mucosa, which may increase and extend gastrin release [12]. Gastroparesis is difficult to assess in children and in patients with significant GI symptoms [56], and cystinosis-specific data are very limited. Younger and smaller children are more likely to have slower gastric emptying [57]. Delayed emptying is also common in kidney failure and dialysis and does not always correlate with GI symptoms [58, 59]. In a study of 50 adult kidney transplant recipients, 48% had gastroparesis, without significant correlations with age, sex, weight, allograft function, or albumin or hemoglobin levels [60]. There may be a role for prokinetic therapy and/or antiemetic neurokinin-1 receptor antagonists when typical treatments for dyspeptic symptoms are ineffective; however, these treatments have not been formally studied in cystinosis [9, 12, 56, 61].

Careful monitoring and adjustments to cysteamine dosing and administration are essential given the likelihood of side effects (Table 4). Abrupt dosage increases and high dosages are associated with GI upset. It is recommended to start patients at a low dosage and increase gradually to avoid intolerance. If symptoms occur, therapy should be interrupted and reintroduced at a lower dosage [33, 34]. For patients switching from IR to DR cysteamine, it is recommended to start at the same dosage as the previous IR cysteamine dosage [34]; in these cases, it is important to ascertain the actual (vs. prescribed) dosage of IR cysteamine to avoid an inadvertent increase in cysteamine exposure upon starting DR therapy [62].

**Table 4:** Clinical strategies for upper GI complications in cystinosis

Monitoring	• Frequent GI symptom history/symptom review [8, 9, 51] • Routine granulocyte cystine level monitoring to assess treatment adequacy/dosage and adherence relative to tolerability [13]
Specialist consultation and referral	• Consider regular gastroenterology evaluations for all patients with cystinosis in addition to ad hoc consultations [39, 51] - Upper GI endoscopy and other assessments/interventions [42] • Referral to specialized dietitian [39] • Pharmacist/polypharmacy consultation [47]
Oral cysteamine considerations	• Consider cysteamine dosage and formulation relative to tolerability and cystine control [33, 34]: - DR cysteamine requires less frequent dosing - Start at low dosage and increase gradually - Avoid abrupt increases in dosage and/or high dosages - In case of GI side effects, consider pausing and restarting at lower dosage - Consider that some patients may not be able to tolerate therapeutic dosage • For enteric-coated DR cysteamine [34]: - Avoid foods and drugs that increase gastric pH within 1 h of administration (e.g., bicarbonate, carbonate) - Consider alternatives to bicarbonate or carbonate base (e.g., citrate) - Consider administration with source of acid (e.g., fruit juice [other than grapefruit juice]) - Avoid high-fat foods close to administration • For IR cysteamine: - Consider administration with meals or directly afterwards; consider milk, potatoes, or other starchy foods; avoid acidic drinks [47]
Upper GI pharmacotherapy considerations	• Consider treatment for upper GI symptoms: - Acid suppression: PPIs, H2RAs,^a^ antacids [9, 11, 12, 47, 51] - Antinausea therapy (e.g., ondansetron) [51] - Appetite stimulation, gastric accommodation, antinausea: cyproheptadine [70] - Prokinetic therapy [9, 12, 56, 61] • For concomitant medications, consider dosage adjustments or therapeutic alternatives to prevent or improve GI side effects; for example [42]: - Immunosuppressive therapy (e.g., change from tacrolimus to cyclosporine)
Potential interventions for severe cases	• Refer to specialists who may consider/implement: - Enteral feeding tube placement [7, 9, 13, 51] - Antireflux surgery (e.g., fundoplication) [2, 9]

*DR* delayed-release, *GI* gastrointestinal, *H2RA* H2 receptor antagonist, *IR* immediate-release, *PPI* proton pump inhibitor

^a^H2RAs may result in decreased levels of cyclosporine [42]

To allow for 12-h (vs. 6-h) dosing, DR cysteamine has an acid-resistant enteric coating designed to bypass the stomach and release active drug in the small intestine at pH 5.5 to 6.5; ensuring an adequately low gastric pH is paramount to avoid premature drug release [63]. It is recommended to administer with water, fruit juice (other than grapefruit juice), applesauce, or berry jelly and avoid administration with drugs that could acutely increase gastric pH (e.g., bicarbonate, carbonate), high-fat foods, and alcohol [34]. Pavloff et al. assessed the drug’s stability with additional foods and determined that enteric coating integrity was maintained with foods at pH < 5.5, including yogurt, pureed bananas and mangos, and pickle juice [64].

Acid-neutralizing or -suppressing therapies, including antacids, histamine-2 receptor antagonists (H2RAs), and proton pump inhibitors (PPIs), may help mitigate cysteamine-induced gastric acid hypersecretion symptoms and improve patient adherence and prognosis (Table 4) [9, 11, 12, 47, 51]. The use of acid-suppressing therapy with DR cysteamine appears to be at odds with the need to maintain sufficiently low gastric pH to avoid early drug release in the stomach. An open-label, single-dose study of 32 healthy adults determined that overall DR cysteamine exposure was similar when administered with water, orange juice, or omeprazole 20 mg plus water [65]. Dohil et al. further demonstrated reductions in cysteamine-induced gastric acid hypersecretion and improvements in GI symptoms among children with cystinosis with concomitant use of omeprazole (*N* = 11) [12] or esomeprazole (*N* = 12) [11]. In both studies, PPI use significantly increased serum gastrin before and after cysteamine administration and significantly decreased gastric acid output [11, 12]. Mean symptom scores decreased significantly after 16 weeks of omeprazole (*P* < 0.001) [12] or esomeprazole (*P* < 0.001) [11], with continued response 8 to 12 months later. The most dramatic improvements occurred in the first 4 weeks of PPI use, and the most common symptoms (nausea, vomiting, anorexia, and pain) were the most profoundly reduced [11, 12]. Cysteamine absorption and plasma concentrations were unaffected by PPI co-administration [11]. Given that PPIs could increase gastric pH to a degree that prematurely degrades the enteric coating of DR cysteamine, additional investigations may be warranted to further understand this apparent contradiction.

Although there is literature on potential associations between PPI use and risk of hypomagnesemia, acute kidney injury, acute interstitial nephritis, incident kidney disease, kidney disease progression, and kidney failure, definitive cause and effect have not been proven [66]. Caution is also needed regarding risk of rebound acid hypersecretion after PPI discontinuation [67] as well as other possible long-term adverse effects, such as osteoporosis, myopathy, and infections due to alterations in gut microflora [42, 66].

Intractable nausea and vomiting may be alleviated by antiemetics such as ondansetron, which is commonly prescribed to patients with cystinosis [51, 68]. However, caution is warranted because constipation is a common side effect of serotonin (5-HT3) receptor antagonists, and use in the setting of electrolyte abnormalities (e.g., low potassium) is associated with risk of arrhythmia [69]. Cyproheptadine also has 5HT-blocking effects in GI smooth muscle, with evidence supporting its effectiveness in promoting appetite and weight gain and improving gastric accommodation and symptoms of dyspepsia, including nausea, vomiting, and abdominal pain, though its use has not been studied in cystinosis [70].

Measures should be taken to mitigate GI side effects of other treatments prescribed to patients with cystinosis (Table 4). For example, nausea and vomiting are common side effects of tacrolimus, and some patients have improved tolerability after switching to cyclosporine [42]. The use of nonsteroidal anti-inflammatory drugs (NSAIDs) with ulcerogenic potential, such as indomethacin [9, 71], may necessitate consideration of alternative options or addition of acid-suppressing therapy. Another approach for poor tolerability is reducing drug dosage or dividing the total daily dose into smaller doses to reduce side effect intensity and duration [42].

Patients with severe symptoms may require more invasive interventions, such as feeding tube placement to support nutrition and drug administration or fundoplication for gastroesophageal reflux [2, 9, 51]. Robust patient/caregiver education about feeding tube hygiene, as well as swift intervention for any suspected problems, is important to prevent complications [51].

### Lower GI complications in cystinosis

As with other GI complications in cystinosis, lower GI symptoms likely result from consequences of cystine deposition, kidney disease, and treatment effects [7, 9, 12]. Considerably less has been published on lower GI sequelae; the prevalence in cystinosis has been reported in only a few cohort and survey studies (Table 1) [9, 10, 14, 16]. Elenberg et al.’s 1998 questionnaire study of pediatric and adult patients reported that among those who underwent formal GI evaluations (*n* = 35), 20% had documented dysmotility (10% of total study population) and 11% had pseudo-obstruction (6% of study population). Among all study participants, 57% reported diarrhea and/or constipation, 50% reported abdominal pain, and 9% reported intermittent abdominal distension [9]. Two small Iranian cohort studies reported abdominal pain and diarrhea in 13% and 26% of children and 42% and 11% of adults, respectively [10, 16]. Gahl et al.’s analysis of 100 adults followed at the NIH (1985–2006) found that 3 of 33 deaths involved bowel perforations [15]. Case reports have described inflammatory bowel disease and irritable bowel syndrome in two patients with cystinosis (aged 6 and 21 years, respectively) [72, 73].

Cysteamine can also play a role in lower GI symptomatology. Product labels for IR and DR cysteamine list abdominal pain and diarrhea among the common adverse events for these products. Both have warnings about GI ulceration and bleeding and recommend monitoring and decreasing dosage for severe GI symptoms [33, 34]. Given cysteamine’s acid-producing effects [12], diarrhea may result from reduced pancreatic enzyme function caused by cysteamine-induced hyperacidity in the lumen [74].

Fibrosing colonopathy has been reported in at least four pediatric and adult patients on DR cysteamine [75]. A component of DR cysteamine’s enteric coating, the methacrylic acid–ethyl acrylate copolymer Eudragit, has been associated with fibrosing colonopathy almost exclusively in young patients with cystic fibrosis who received Eudragit-coated pancreatic enzymes at extremely high dosages far above recommendations [76]. The FDA considered the risk of fibrosing colonopathy during their review and approval of DR cysteamine and concluded, based on long-term animal toxicity studies, that there is a reasonable assurance of safety for the maximum daily intake of Eudragit with DR cysteamine [77]. Nonetheless, patients on DR cysteamine should be evaluated for severe, persistent, and/or worsening symptoms (e.g., abdominal pain, vomiting, bloody or persistent diarrhea, fecal incontinence); if fibrosing colonopathy is confirmed, the patient should be switched to IR cysteamine [34].

Careful monitoring, counseling, and adjustments to cysteamine dosing and administration are essential given the likelihood of GI effects, and product-specific guidance should be followed to prevent and minimize intolerance. If treatment-emergent lower GI side effects occur, cysteamine should be interrupted and restarted at a lower dosage [13, 33, 34, 50].

The use of NSAIDs, steroids, and immunosuppressants can also contribute to lower GI complications, such as ulcers and perforations [42], which can be deadly but appear to be relatively uncommon in patients with cystinosis, especially in the CDT era [15]. For other medications prescribed to patients with cystinosis, such as immunosuppressive therapy, consider adjusting dosage or switching to alternative treatments with fewer GI side effects, if appropriate [42]. A 2004 systematic review of mycophenolate mofetil (MMF) versus azathioprine following kidney transplant found that the acute and chronic incidence of diarrhea was higher with MMF [78]. Slowed colon cell regeneration, increased apoptosis, and compromised villous structure of the duodenum may contribute to the elevated risk of MMF-associated intestinal side effects [42]. MMF is also associated with increased incidence of bowel perforations on long-term follow-up [79]. For these reasons, MMF is sometimes replaced with enteric-coated formulations or other immunosuppressive options with fewer GI side effects [80].

Early recognition and aggressive treatment of lower GI problems can improve or prevent disabling complications [9]. It is important to regularly ask patients about lower GI symptoms, with a low threshold for specialist consultation/referral (Table 5). Even suspected mild GI events should be investigated aggressively given the potential for life-threatening progression and the impact of GI symptoms on adherence to the prescribed treatment regimen [39, 42, 51]. Referral to a motility specialist/clinic may help in some cases, such as when standard interventions for diarrhea or constipation are ineffective [81].

**Table 5:** Clinical strategies for lower GI complications in cystinosis

Monitoring	• Frequent GI symptom history/symptom review [8, 9, 51] • Routine granulocyte cystine level monitoring to assess treatment adequacy/dosage and adherence relative to tolerability [13]
Specialist consultation and referral	• Consider regular gastroenterology evaluations for all patients with cystinosis in addition to ad hoc consultations [39, 51] - Aggressive investigation of suspected GI events [42] - Consider further assessment such as endoscopy/colonoscopy, biopsy [42] - Refer to motility specialist/clinic, as needed [81] • Referral to specialized dietitian [39] • Pharmacist/polypharmacy consultation [47]
Oral cysteamine considerations	• Consider cysteamine dosage and formulation relative to tolerability and cystine control; per drug labeling [33, 34]: - Start at low dosage and increase gradually - Avoid abrupt increases in dosage and/or high dosages - Monitor closely for GI toxicity; pause and restart at lower dosage
Lower GI pharmacotherapy considerations	• For concomitant medications, consider dosage adjustments and therapeutic alternatives to prevent or improve GI side effects; for example [42]: - Immunosuppression: consider dividing total daily dose into smaller doses or decreasing total dosage, if possible - MMF: consider enteric-coated option or alternative (e.g., azathioprine) - Steroids: consider withdrawal in cases of serious GI complications, depending on strength of first-line immunosuppressive therapy - NSAIDs: consider risks in context of concomitant medications that impair intestinal tract cytoprotection

*GI* gastrointestinal, *MMF* mycophenolate mofetil, *NSAID* nonsteroidal anti-inflammatory drug

### Hepatic complications in cystinosis

Hepatomegaly without liver failure is relatively common in cystinosis, typically characterized by nodular regenerative hyperplasia without accompanying fibrosis but sometimes causing splenomegaly and portal hypertension [5, 7, 8]. Less common findings include cholestasis, varices, and ascites [5, 10, 82–85]. Liver biopsies from case reports and small cohort studies have shown significant cystine deposition in Kupffer cells, presumed to be the cause of hepatomegaly and liver-protein synthetic dysfunction in cystinosis [7, 82, 83, 86–88].

In CRN’s 2016 survey of adults with cystinosis, 5% reported liver disease [14]. Small cohort studies of children and adults have reported rates of hepatomegaly, splenomegaly, and increased portal vein velocity of up to 74%, 53%, and 58%, respectively (Table 1) [8, 10, 16]; rates of abnormal liver enzymes, portal hypertension, cholestasis, and ascites were all < 10% [8, 10, 16, 83, 89].

Hepatic sequelae appear to be less frequent with the advent of oral cysteamine [5]. Brodin-Sartorius et al.’s 2012 analysis of 86 patients with cystinosis (mean age, 27 years) suggested that hepatosplenic complications have become increasingly rare; 21% of patients underwent splenectomy due to portal hypertension, but none were performed after 1997 [6]. Topaloglu et al.’s 2020 study of 21 children in Turkey (mean age, 11 years) reported that 74% had hepatomegaly and 53% had granular pattern or heterogeneity of the liver. Ten patients (53%) had splenomegaly, and 58% had increased portal vein velocity. Elevated liver enzymes were noted in 5% of patients [8]. A 2022 study of 19 adults with cystinosis in Iran (mean age, 24 years) reported that 21% had hepatomegaly and splenomegaly; one patient had increased portal vein flow velocity, and none had elevated liver enzymes [16].

 Patients with cystinosis should be monitored regularly for liver complications (Table 6). International consensus-based expert guidance published in 2022 recommends annual systemic liver function and lipase testing to monitor for hepatic and pancreatic dysfunction in adolescent and adult patients [39]. Signs of hepatomegaly or splenomegaly warrant specialist referral [39]. Patients with splenomegaly or increased portal vein diameter or flow should be followed in case of progression to portal hypertension [8]. Two case reports including three patients (aged 26, 29, and 36 years) have described interventions for portal hypertension and cholestasis in cystinosis [82, 88].

**Table 6:** Clinical strategies for hepatic complications in cystinosis

Monitoring and specialist coordination	• Assess liver and pancreas function annually [5, 39] - AST, ALT, GGT, ALP - Lipase • Collaborate with gastroenterologist or hepatologist for further assessment [39] - Ultrasound for signs of hepatomegaly and/or splenomegaly - Close follow-up of patients with splenomegaly and/or increased portal vein diameter [8]
Potential interventions for advanced disease	• Refer to specialists who may consider or implement: - Portosystemic shunting with aggressive medical therapy for noncirrhotic portal hypertension [36] - Ursodeoxycholic acid for cholestatic liver disease [82]

*ALP* alkaline phosphatase, *ALT* alanine transaminase, *AST* aspartate transaminase, *GGT* gamma-glutamyl transferase

## Summary and conclusions

Cystine-depleting therapy and advances in kidney transplantation have transformed the course of cystinosis from a fatal kidney disease to a manageable lifelong condition, tasking nephrologists with addressing sequelae beyond the kidneys. GI complications in cystinosis are highly prevalent and multifactorial, representing a significant clinical challenge. Given the profound impact of GI symptoms and the complexity of untangling and addressing their many causes, three key imperatives emerged from this review and discussion:

1. Positive inquiry: routine, proactive GI symptom history-taking, and symptom review with patients and caregivers.
2. Polypharmacy management: careful consideration of treatment formulations, safety profiles, interactions, dosage adjustments, timing, and therapeutic alternatives to optimize tolerability, adherence, and disease control.
3. Multidisciplinary collaboration: regular consultation and referral with gastroenterology, pharmacy, and other specialists for evaluations, interventions, and co-management.

Nephrologists, who typically assume the bulk of cystinosis management, should galvanize their wider team and enlist specialists to help co-manage these patients. Despite high GI symptom burden, it has been reported that < 20% of adults with cystinosis are under the care of a gastroenterologist [14]. Regular and ad hoc gastroenterology consultations can help attenuate GI complications in pediatric and adult patients. Pharmacists can serve as invaluable resources for complex medication management. Providing patients and caregivers education and resources about GI complications in cystinosis will benefit patients directly and help them inform other providers, such as primary care and adult specialist physicians, who may not be familiar with the disease [14]. Nephrologists should also share relevant disease background and context with specialists, such as gastroenterologists, who are tasked with helping address extra-renal complications in patients with cystinosis.

There are many gaps to fill in our understanding and treatment of GI sequelae in cystinosis. Larger, more robust studies are needed to assess prevalence and risk factors for various GI complications, including investigations into how the incidence and severity of these symptoms are impacted by differences in treatment and markers of disease control. The efficacy of various interventions to mitigate GI complications in patients with cystinosis should also be evaluated, for example, directly measuring gastric pH during DR cysteamine dosing, comparing the effects of PPI versus H2RA co-administration, and assessing outcomes of other potential supportive treatments like cyproheptadine. Given the growing evidence on the role of inflammation in cystinosis, preexisting or novel anti-inflammatory therapies may improve GI complications [24], but studies are also needed in this area.

Investigational cystinosis therapies, such as hematopoietic stem cell gene therapy, currently in phase 1/2 trials [90], aim to prevent cystine-induced damage and eliminate the need for long-term treatments altogether. However, the potential benefits of new therapies must be balanced with their inherent risks. In the meantime, given the profound impact of GI symptoms on patient well-being, treatment adherence, and therapeutic success, clinicians must work across disciplines to proactively address GI concerns in cystinosis rather than accepting them as inevitable consequences of the disease and its treatments.

## Declarations

### Disclaimer

The authors confirm independence from the sponsor; the content of the article has not been influenced by the sponsor. No authors received compensation for the preparation of this manuscript.
